# The Second Asia-Oceania Human Proteome Organization (AOHUPO) Online Education Series on the Renaissance of Clinical Proteomics: Biomarkers, Imaging and Therapeutics

**DOI:** 10.1016/j.mcpro.2022.100436

**Published:** 2022-10-26

**Authors:** Teck Yew Low, Yu-Ju Chen, Yasushi Ishihama, Max Ching Ming Chung, Stuart Cordwell, Terence Chuen Wai Poon, Ho Jeong Kwon

**Affiliations:** 1 UKM Medical Molecular Biology Institute (UMBI), Universiti Kebangsaan Malaysia, Kuala Lumpur, Malaysia; 2 Institute of Chemistry, Academia Sinica, Taipei, Taiwan; 3 Graduate School of Pharmaceutical Sciences, Kyoto University, Kyoto, Japan; 4 Department of Biochemistry, Yong Loo Lin School of Medicine, National University of Singapore, Singapore; 5 School of Life and Environmental Sciences and Sydney Mass Spectrometry, The University of Sydney, Sydney, Australia; 6 Pilot Laboratory, Proteomics Core, Institute of Translational Medicine, Centre for Precision Medicine Research and Training, Faculty of Health Sciences, University of Macau, Macau SAR, China; 7 Chemical Genomics Leader Research Initiative, Department of Biotechnology, Yonsei University, Seoul, South Korea

## Abstract

In 2021, the Asia-Oceania Human Proteome Organization (AOHUPO) initiated a new endeavor named the AOHUPO Online Education Series with the aim to promote scientific education and collaboration, exchange of ideas and culture among the young scientists in the AO region. Following the warm participation, the AOHUPO organized the second series in 2022, with the theme “**The Renaissance of Clinical Proteomics: Biomarkers, Imaging and Therapeutics**”. This time, the second AOHUPO Online Education Series was hosted by the UKM Medical Molecular Biology Institute (UMBI) affiliated to the National University of Malaysia (UKM) in Kuala Lumpur, Malaysia on three consecutive Fridays (11th, 18th and 25th of March). More than 300 participants coming from 29 countries/regions registered for this 3-days event. This event provided an amalgamation of six prominent speakers and all participants whose interests lay mainly in applying MS-based and non-MS–based proteomics for clinical investigation.

## Highlights

• The AOHUPO organized the second AOHUPO Online Education Series in March 2022.
• An amalgamation of speakers and participants in clinical proteomics was provided.
• The AOHUPO continues this event online that brings AO researchers together.

## Graphical Abstract

**Figure:**
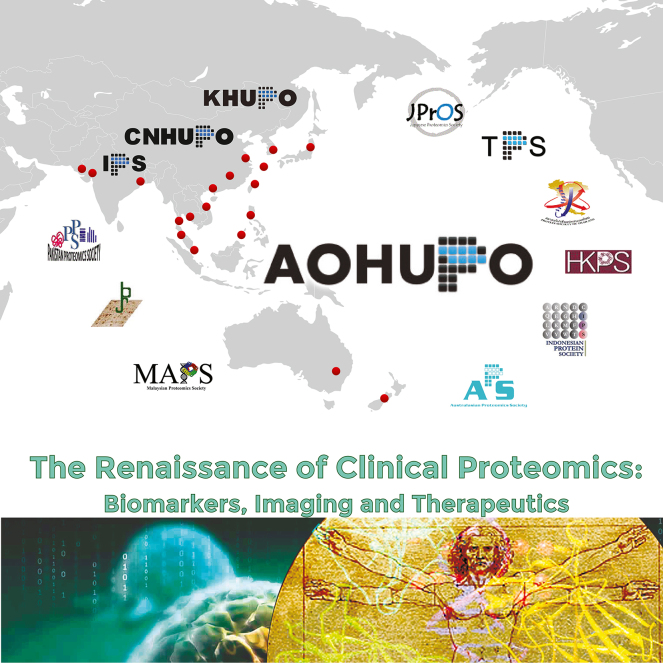

## In Brief

In 2021, the Asia-Oceania Human Proteome Organization (AOHUPO) initiated the AOHUPO Online Education Series (AOHUPO OES) with the aim to promote scientific education and collaboration and exchange of ideas and culture among the young scientists in the AO region. Following the warm participation, the AOHUPO organized the second series in 2022, with the theme “The Renaissance of Clinical Proteomics: Biomarkers, Imaging and Therapeutics.” This event provided an amalgamation of six prominent speakers and all participants whose interests lay mainly in applying MS-based and non-MS-based proteomics for clinical investigation.

The Asia-Oceania Human Proteome Organization (AOHUPO; http://www.aohupo.org/) was established in 2001 to promote and coordinate the activities of the 17 participating regional proteomics societies (1). In 2021, the AOHUPO Online Education Series (AOHUPO OES) was launched to encourage scientific education and collaboration among young proteomics researchers. The first AOHUPO OES was entitled “***Next-generation Proteomics in Precision Oncology***” organized by the University of Macau, which received active participation from the AO regions. Following its success, the AOHUPO organized the second AOHUPO OES in 2022, with “***The Renaissance of Clinical Proteomics: Biomarkers, Imaging and Therapeutics***” as its theme.

**Fig. 1:**
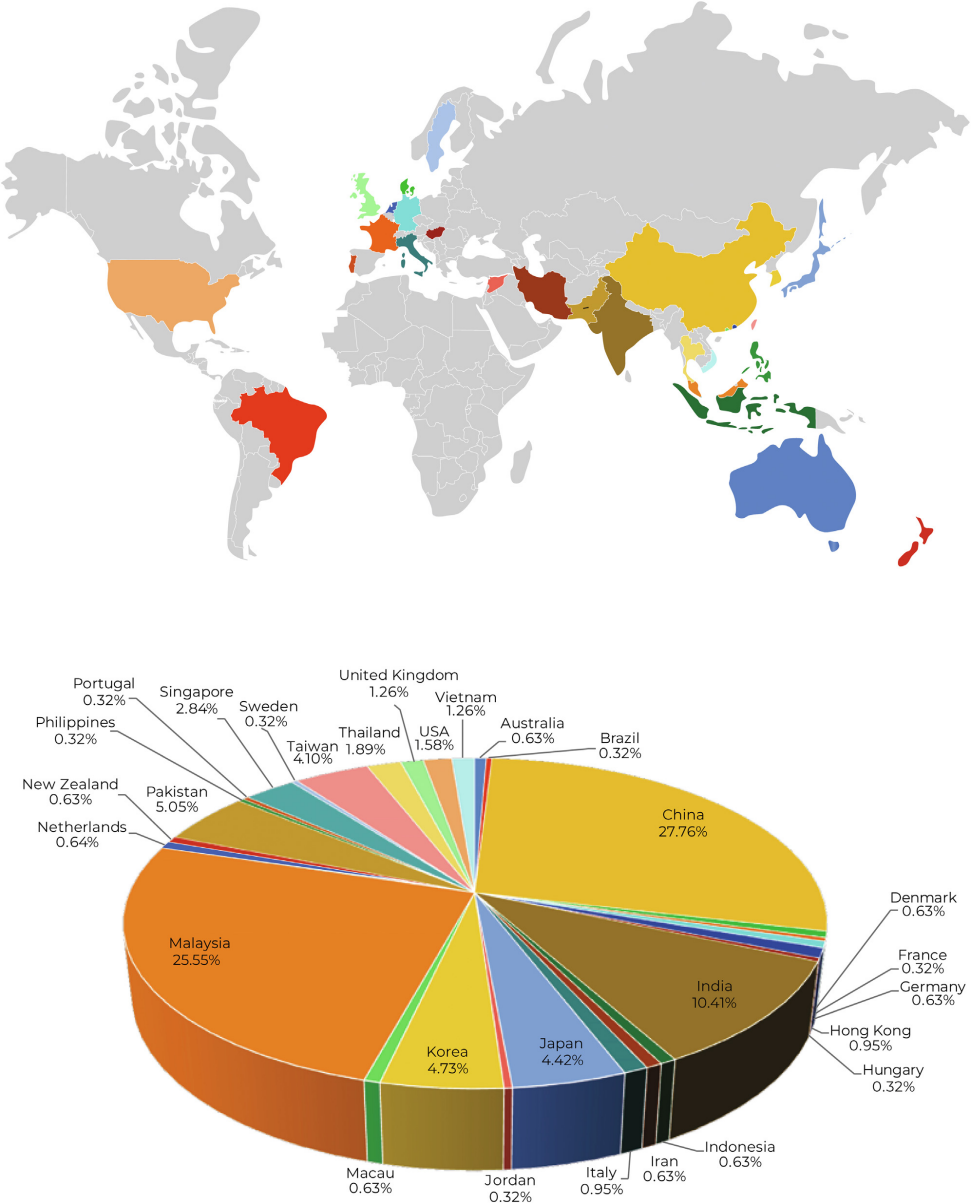
**Registration statistics according to countries/geographical regions for the second AOHUPO Online Education Series 2022**.

The second OES comprised three 2.5-h webinars on three Fridays (11th, 18th and 25th March 2022), hosted by the UKM Medical Molecular Biology Institute in Malaysia. The organizing committee received registrations from 334 participants who came from 29 countries/regions (Fig. 1). Almost 70% of registrants comprise postgraduate students and post-docs, the rest came from the academia and industry. Six prominent international speakers were invited, covering different aspects of clinical proteomics. All talks were followed by a Q&A session in a forum format. Besides the two cochairs, several AOHUPO councils formed the discussion panels and questions were taken live from the audience. To forge close collaboration with the HUPO, three HUPO-ECRs, that is, Dr Mia Iwasaki, Dr Dongxue Wang, and Dr Jong Seo Kim, were also invited as panellists.

**Fig. 2:**
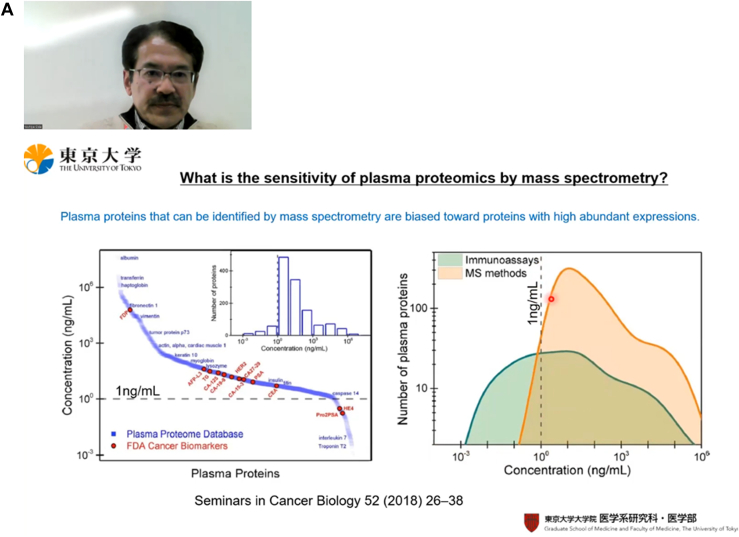

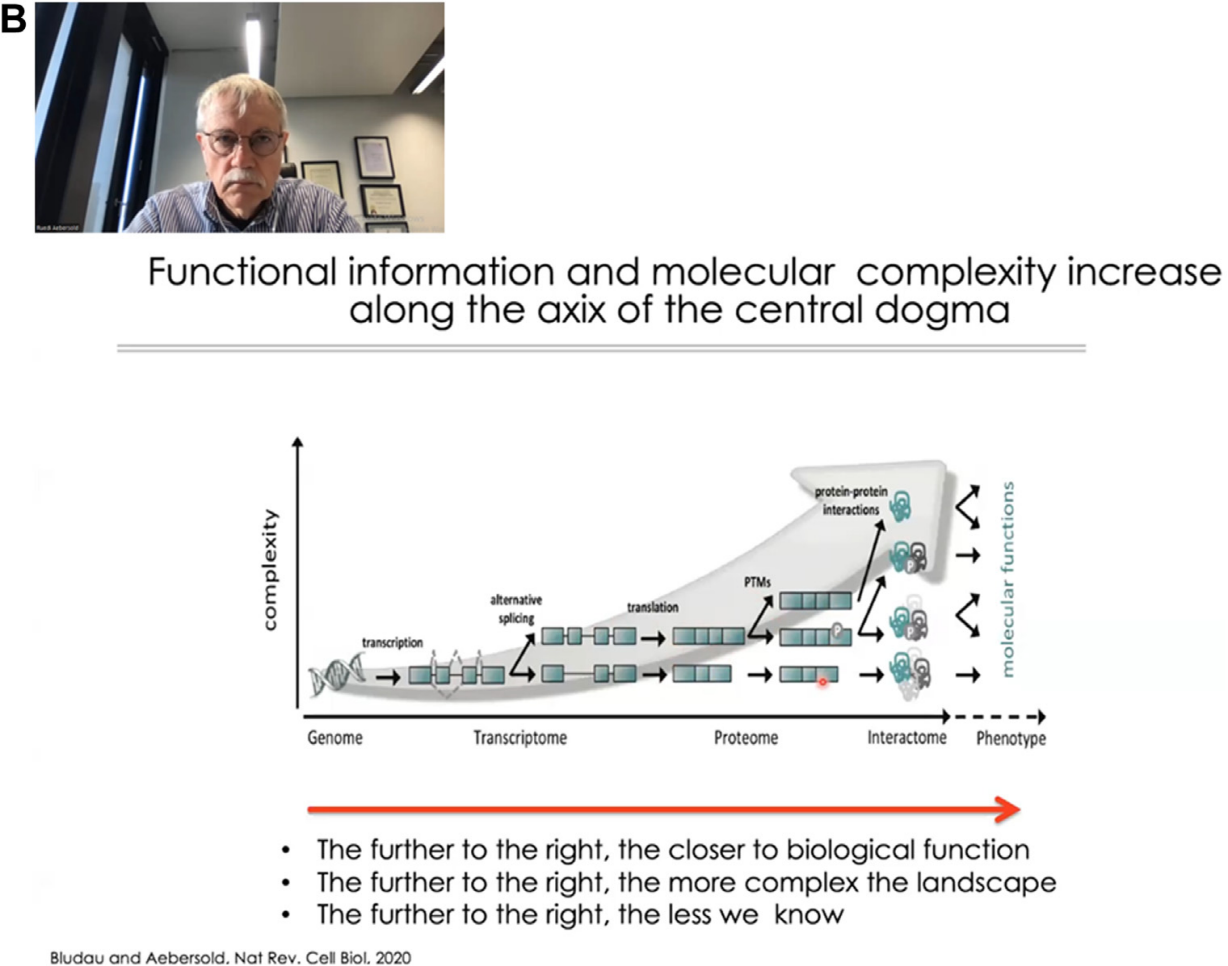
**Photos of two plenary talks on the first day (March 11) of the event.** (*A*) Professor Yoshiya Oda from the University of Tokyo presenting “The Challenge of Hypothesis Building from Multi-Omics: The Case of Alzheimer's Disease”, followed by (*B*) Professor Ruedi Aebersold from ETH Zurich’s talk on “Exploring the Uncharted Proteome”.

**Fig. 3:**
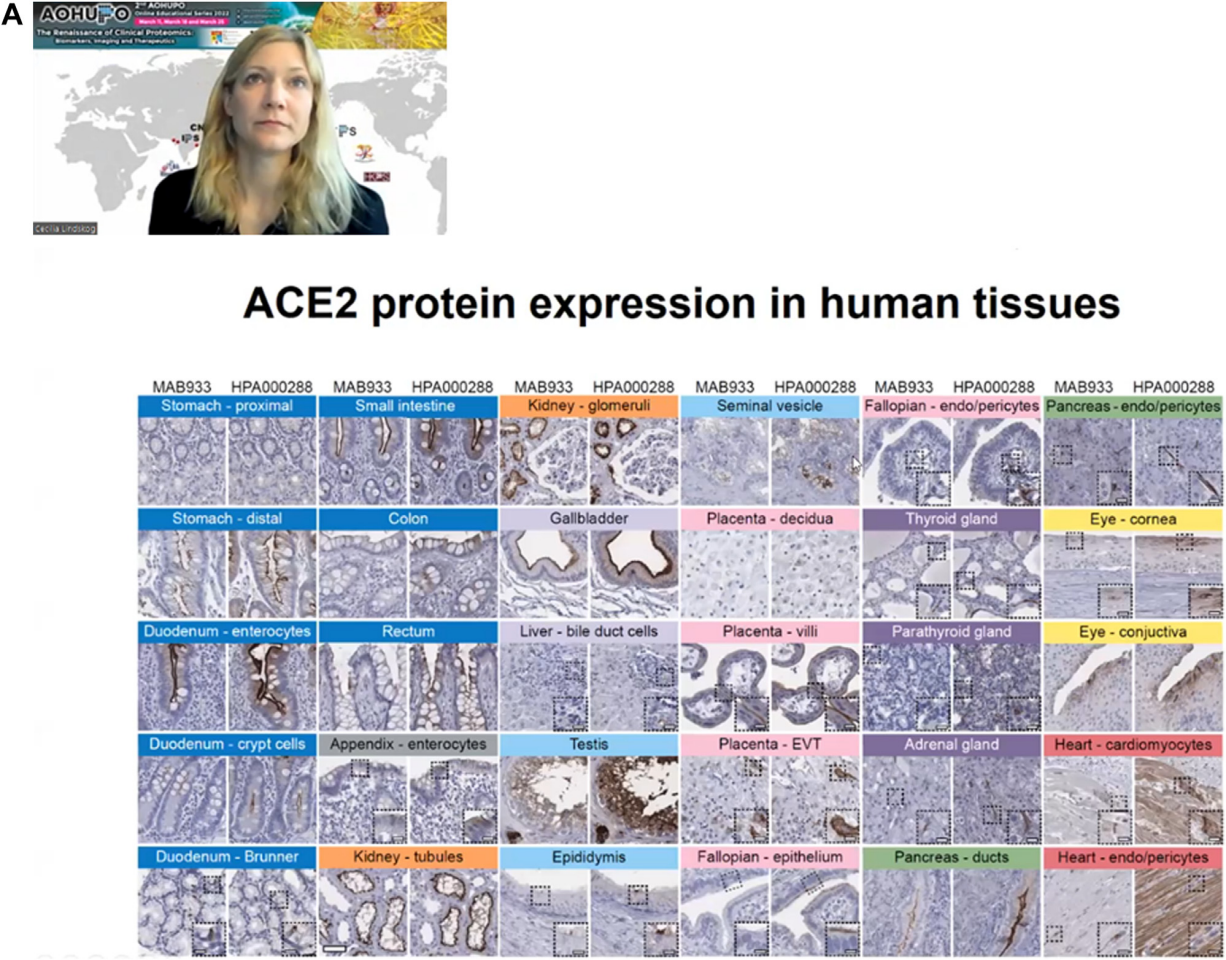

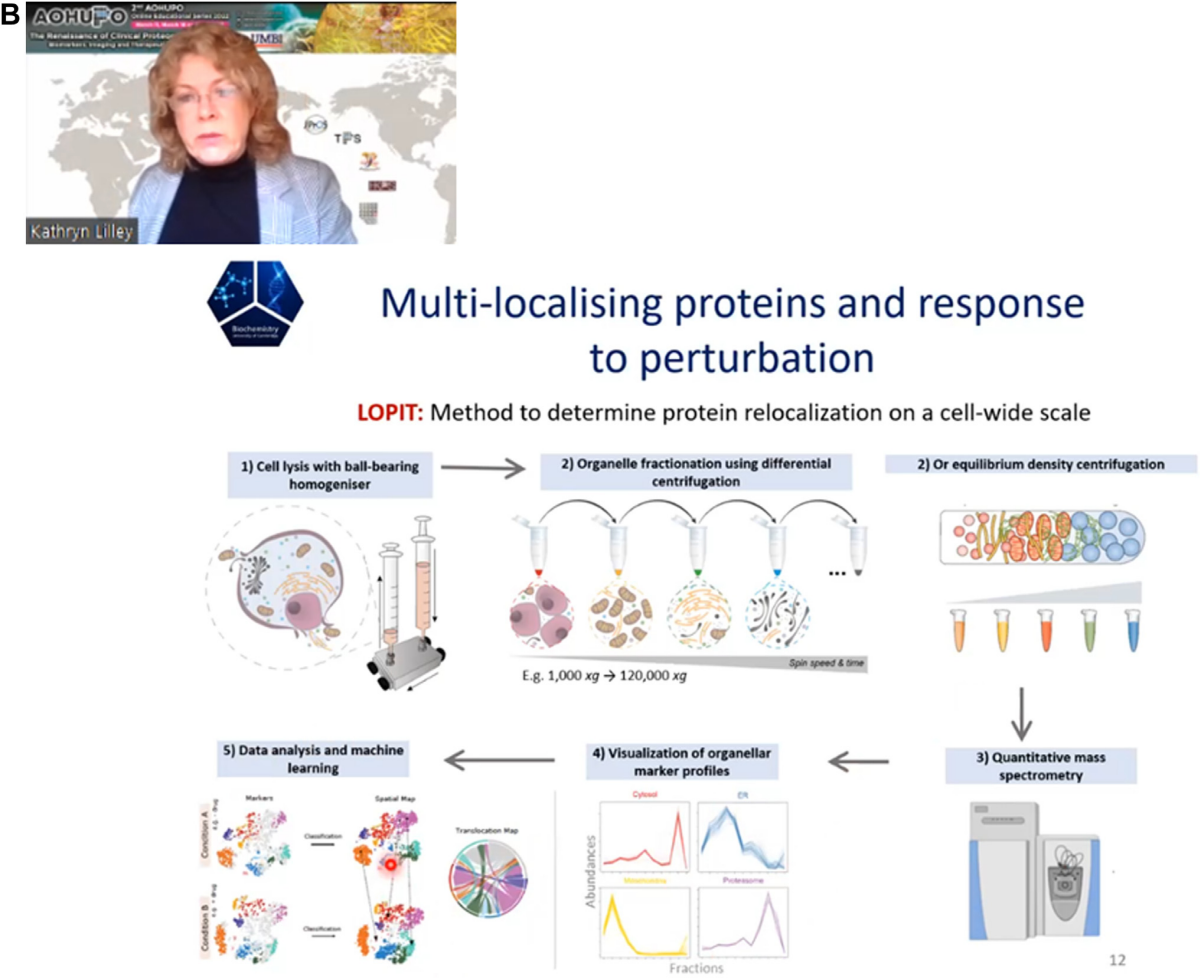
**Photos of two plenary talks on the second day (March 18) of the event.** (*A*) Dr Cecilia Lindskog from Uppsala University presented “The Human Protein Atlas – spatial proteomics in health and disease”, focusing on the Human Tissue Atlas. (*B*) ) Next, Professor Kathryn Lilley, a pioneer in spatial proteomics gave a presentation on “The Spatial Organization of the Cell”.

**Fig. 4:**
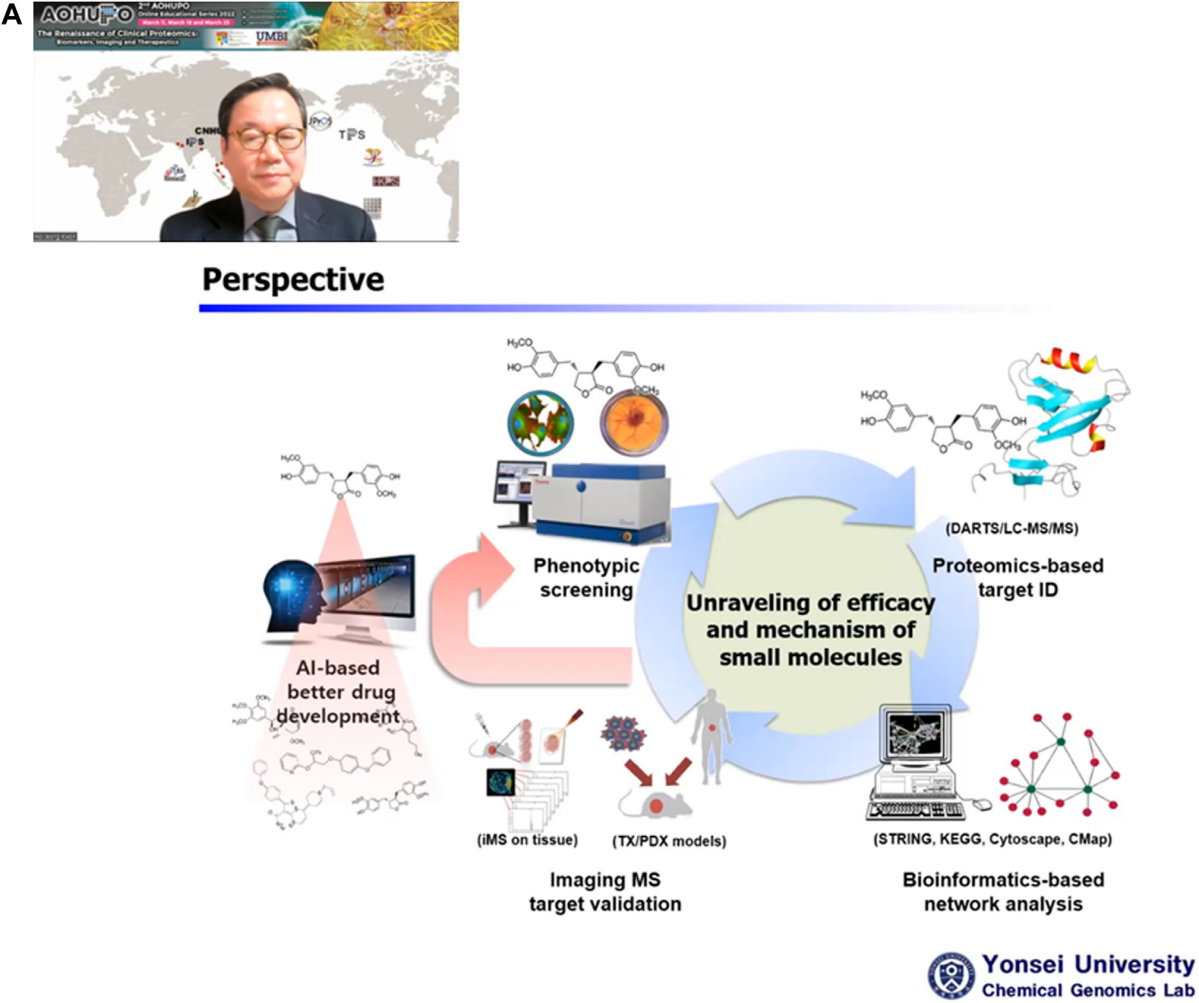

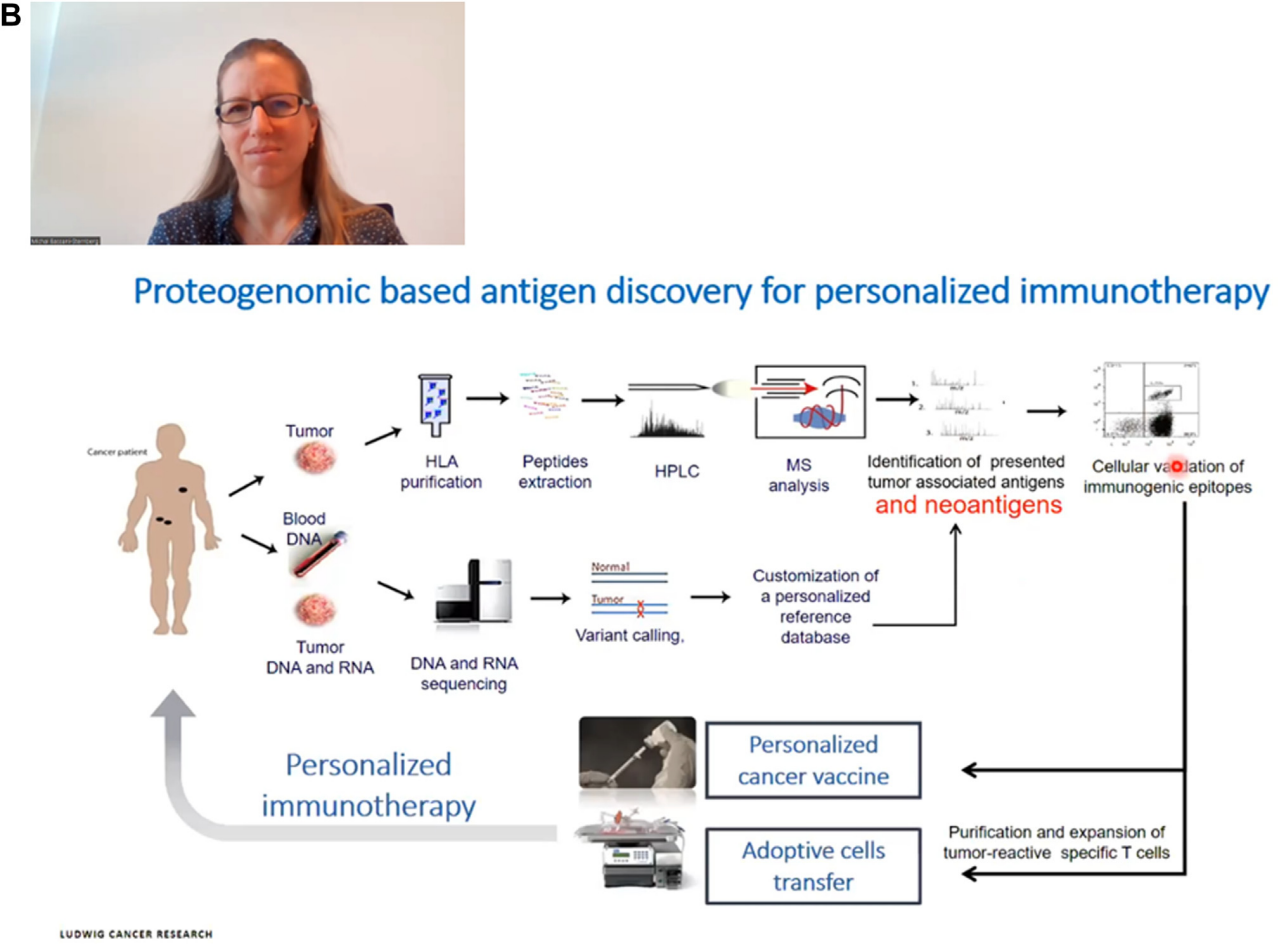
**The last day for the second AOHUPO OES on therapeutics.** The day started with (*A*) Professor Ho Jeong Kwon from Yonsei University who presented “Exploring Drug-Target-Phenotype Interaction with MS-based Proteomics Approaches and its Translational Impacts”. (*B*) This was followed by Dr Michal Bassani-Sternberg’s plenary session titled “Identification of HLA ligands through MS-based Immunopeptidomics for Development of Personalized Cancer Immunotherapy”.

## DAY 1: 11th MARCH 2022

The second AOHUPO OES was jointly open by Professor Ho Jeong Kwon, the President of AOHUPO, and Associate Professor Nor Azian Abdul Murad, the director of UKM Medical Molecular Biology Institute; both also acted as cochairs for Professor Yoshiya Oda (University of Tokyo)’s plenary talk entitled “**The Challenge of Hypothesis Building from Multi-Omics: The Case of Alzheimer's Disease**”.

Professor Oda first introduced proximity extension assay (PEA), which is more sensitive in accessing the low-abundance portion of the plasma proteome (<1 ng/ml) than LC-MS (2, 3). He demonstrated its high coverage in detecting and quantifying low-abundance cytokines, besides its superiority in throughput, reproducibility, and linear dynamic range. Interestingly, he found no overlap between PEA and LC-MS datasets acquired from plasma samples. He next presented an in-house–developed tandem mass tags-lipidomics technique that can label diverse classes of lipids (4, 5). These techniques were then combined to investigate the plasma samples from elderly Japanese with different degrees of Alzheimer’s disease (AD). Proteome data from PEA and LC-MS, though having little overlap, were enriched in chemokine and platelet activation pathways, respectively; implying platelet activation by vascular damage and subsequently induction chemokine release are associated with AD. Independently, tandem mass tags-lipidomics data demonstrated the upregulation of bile acids and the downregulation of docosahexaenoic acid/eicosapentaenoic acid)-phospholipids and docosahexaenoic acid/eicosapentaenoic acid-triglycerides in AD plasmas. This led to the hypothesis that, in AD, vascular aging and damage results in leaky blood vessels at the intestinal barrier as well as the blood brain barrier. Consequently, toxins leak from the intestines into the blood, which are then transferred to blood brain barrier (Fig. 2).

Many participants were intrigued. Among the questions raised include how such multi-omics study can assist treatment/prevention of AD, and if existing drugs for treating blood vessel leakage would be effective for treating AD. Another panellist, Professor Maxey Chung wondered if the little overlap between LC-MS and PEA datasets would affect final data integration. Meanwhile, a young scientist from Malaysia was interested to know if a longitudinal study was available. Further, since blood vessel leakage is often linked to inflammation, this might point the likely association of AD with inflammation. Prof. Oda concluded that multi-omics integration of AD is still at an early stage, whereby the main bottleneck arises from sample availability which constraints longitudinal. Also, PEA is specifically designed as panels that target a certain selection of low-abundance proteins; therefore, it can be biased. On the other hand, LC-MS adopts a discovery approach that tends to detect higher abundance proteins. Therefore, both methods should be viewed as complementary.

Professor Ruedi Aebersold (ETH Zurich) delivered the second talk - “**Exploring the Uncharted Proteome**”. Proteins are the closest surrogates to functional phenotypes, though contemporary proteomics research falls short of proteome-wide investigation of the proteoforms and protein-protein interaction (PPI) networks (6). Furthermore, most PPI studies do not reflect context-specific and dynamic changes of PPI. As a solution, Prof. Aebersold’s lab developed “SEC-SWATH-MS”, combining size-exclusion chromatography to data-independent MS acquisition (DIA) (7). With this technique, mildly extracted protein complexes are SEC-separated and analyzed with SWATH-MS. An algorithm called SECAT was developed to analyze the comigration profile of protein complexes (8). Applying these methods on two HeLa cell variants which are susceptible (CCL4) and resistant (Kyoto) to *Salmonella typhimurium* infection, his lab ascribed the resistance of the HeLa Kyoto line to its failure in forming mature invadopodium as a result of missing WIPF1 or WPF2 proteins. Prof. Aebersold next described another algorithm named “COrrelation based ProteoForms Assignment” (COPF), which mines existing bottom-up DIA datasets to reveal the alternative spliceforms among different conditions (9). It does this by first measuring peptide intensity across samples, followed by pairwise correlation, clustering, and proteoform scoring. COPF can be used in combination with SEC-SWATH-MS to reveal cell state–specific proteoforms forming different protein complexes (9) (Fig. 2).

Among the interesting questions from the audience include (i) how proteolytic digestion and its effects on protein coverage affect the detection of proteoforms and whether (ii) digestion with multiple proteases would improve the COPF algorithms. Prof. Aebersold explained that while it would be ideal (but rarely) to achieve 100% protein coverage, the use of DIA improves the reproducibility of peptide detection such that if a missing peptide is detected in a given sample, it can be used to formulate a new hypothesis and to test the presence of a new proteoform—be it phosphorylated, spliced, out or carrying a mutation.

## DAY 2: 18th MARCH 2022

On the 18th of March, Dr Cecilia Lindskog (Uppsala University) presented “**The Human Protein Atlas – spatial proteomics in health and disease**”, introducing The Human Protein Atlas (HPA; www.proteinatlas.org/). She focused on the human Tissue Atlas, which contains immunohistochemistry images of tissue microarrays in single cell resolution, as well as quantitative transcriptomics data for each protein-coding gene (10). Currently, the Tissue Atlas covers 15,323 genes (76%), their localization in 78 different cell types and 44 tissue types, supported by quantitative bulk transcriptomics data for 37 normal human tissue types acquired in-house (HPA 37 tissues and 18 blood cell types) from GTEx (36 tissues) and FANTOM5 (60 tissues). To define the tissue-specify of all human genes, the expression levels and spatial distribution of mRNAs were divided into five enrichment categories. Recently, UMAP gene clustering was also made available for viewing the expression patterns of the nearest gene neighbors per tissue type. The latest version (Ver. 21) of the Tissue Atlas additionally incorporates single-cell transcriptomics (sc-RNA-seq) data. Up to now, sc-RNA-seq data covers 25 human tissues and blood cells, including 444 single cell type clusters and 76 main cell types. Recently, Dr Lindskog used this resource to study the human angiotensin I converting enzyme 2 (ACE2), a cell surface receptor involved in host cell entry for SARS-CoV2 (11). Bulk transcriptomics showed that ACE2 is expressed in abundance in small intestines, colon, and duodenum but not in the lungs, corroborated by sc-RNA-seq data obtained from various constituent cell types. This observation was validated by immunohistochemistry performed with two different antibodies targeting ACE2 of 45 normal tissue types and 159 different cell types from ∼800 individuals (Fig. 3).

The session cochair Prof. Hsueh-Fen Juan was particularly surprised that ACE2 was expressed only at low levels in the lungs although it is a receptor for SARS-CoV2. She further questioned if Dr Lindskog had plan to also investigate tissue-specific microbiome. According to Dr Lindskog, SARS-CoV2 had been shown to first enter human body *via* the upper airway including the nose and bronchus before it enters the lungs. Meanwhile, investigation of the microbiome is still not a part of the HPA. Another audience inquired if there are highly expressed mRNAs or proteins that do not correlate with the corresponding tissue or organ functions. Dr Lindskog concurred and informed that HPA did find mRNAs and proteins that do not agree with their functions as documented in the UniProt; and research is still underway to resolve this discrepancy. The President of HUPO, Prof. Yu-Ju Chen commented that HPA is a useful resource used by many colleagues for their research and publication. She hoped that Dr Lindskog can organize a HPA training course for this region, which Dr Lindskog gladly agreed.

Professor Kathryn Lilley (University of Cambridge) presented “**The Spatial Organization of the Cell**”. Proteins and RNAs must traffic to the correct cellular localities for proper functions; whereas aberrant localizations may end up in diseases. Prof. Lilley studies how proteins/RNAs end up at the right addresses; how biomolecular features affect their localities, and whether proteins/RNAs carry out the same functions at different locations. Her lab develops methods to characterize the localizations of proteins and RNA in a system-wide manner. To assign the proteome to correct subcellular addresses, an ultracentrifugation-based subcellular fractionation method called LOPIT (Localization of Protein using Isotope Tagging) is used, where similar correlation profiles of proteins among the consecutive ultracentrifuged fractions indicate organelle colocalization (12). LOPIT can be achieved by equilibrium density gradient ultracentrifugation (HyperLOPIT) offering superior resolution or differential ultracentrifugation (LOPIT-DC), which required less starting materials (13, 14). Function-wise, LOPIT can be used to generate a “static map” or a “dynamic map”. While a static map is typically employed to assign protein to a subcellular locality under a specific condition or to identify new organelles or poorly characterized subcellular complexes, a dynamic map is used to probe the specific relocalizations of proteins upon cellular perturbations, diseases, or developmental stages. In a recent study, THP-1 monocytes were treated with lipopolysaccharide to induce inflammatory response, followed by HyperLOPIT to generate a dynamic map (15). Interestingly, among the 253 relocalized proteins, only two registered changes in abundance, implying that measuring protein abundance alone is fallible. Finally, the two LOPIT protocols were adapted for investigating the (re)localization of the transcriptome, giving raise to LoRNA (Localization of RNAs), which aims to assign the transcriptome including both coding and noncoding RNAs to all subcellular localities (16). Both LOPIT and LoRNA can be performed in a single-step subcellular fractionation to simultaneously localize proteins and RNAs (Fig. 3).

The discussion panel and audience raised several questions to this thought-provoking topic, including (i) how LOPIT and LoRNA can be used to study neurodegenerative diseases; (ii) how RNA binding can contribute to the relocalization of proteins; and (iii) the possibility of RNAs acting as a molecular chaperone to assist protein folding. Prof. Lilley explained that RNA–protein interaction is a widely observed phenomenon, including many metabolic enzymes. However, it is still early to tell if the functions of such RNA-protein are linked or that they serve other moonlighting purposes.

## DAY 3: 25th MARCH 2022

In his plenary talk entitled “**Exploring Drug-Target-Phenotype Interaction with MS-based Proteomics Approaches and its Translational Impacts**”, Professor Ho Jeong Kwon (Yonsei University) described how he chose small molecules as tools to explore the functional proteome for therapeutic application. The key strategy in his lab involves in a step-wise manner: (i) cell-based phenotypic screen with chemical libraries to identify target small molecules that can perturb a selected phenotype; (ii) chemical proteomics to identify protein targets that bind to these target small molecules; (iii) validation of drug-protein binding with structural bioinformatics and activity and functional assays; (iv) designing better drugs and establishing platform to expedite drug discovery; thus providing (v) feedback to discover new drugs targeting new biological process (17). He elaborated a procedure for discovering small molecules targeting autophagy, a self-degradation process which is related to cancers and neurodegenerative diseases. In this process, his lab first performed phenotypic screen with chemical libraries against HeLa cells, followed by Acridine Orange staining which detects lysosome activity (18). This resulted in the discovery of several putative autophagy inducers including Kaem, Sert, Rg3, and CTS (19). To identify target proteins of these small molecules, his lab adopted label-free chemical proteomics methods such as DARTS (Drug Affinity Responsive Target Stability) and CETSA (Cellular Thermal Shift Assay) (20). Both methods are based on the premise that drug binding confers conformational change, as well as protease and heat stability to a protein, rendering it less susceptible to proteolysis (DARTS) and heat denaturation (CETSA) relative to the unbound population. The read-outs for these changes can be registered by comparing the drug-treated and untreated proteome using western-blot or quantitative MS-based proteomics. Finally, he finished the talk by explaining how imaging MS can be utilized to localize the distribution of small molecule distribution in living tissues, and how these data can be collectively analyzed by artificial intelligence to design better drugs (21) (Fig. 4).

Prof. Shamshad from Pakistan commented that Prof. Kwon workflow was unique as most similar studies would start with computational simulation such as molecular docking before wet lab. Prof. Maxey Chung questioned about the chemical libraries used. According to Prof. Kwon, he prefers to first use wet lab approach to identify real drug targets using proteomics before validating them with computational approaches, if protein structures are available. He also mentioned that the quality of a chemical library is essential. In his case, he prefers a knowledge-driven approach in selecting lead compounds that have well-known traditional efficacy and are sourced from the local environment but with unknown protein targets.

In the last plenary talk - “**Identification of HLA ligands through MS-based Immunopeptidomics for Development of Personalized Cancer Immunotherapy**”, Dr Michal Bassani-Sternberg (Ludwig Institute of Cancer Research in Lausanne) introduced MS-based immunopeptidomics for tumor antigen discovery, in unraveling the tumor-associated antigens (22). The HLA is both polygenic and polymorphic, with each form binding to antigenic peptides carrying defined sequence motifs, which can now be computationally predicted. Her lab had established a workflow which immunopurifies sequentially, both HLA-I and HLA-II peptides for data-dependent acquisition- and data-independent acquisition-based MS acquisition (23). To detect tumor-specific neoantigens, her lab incorporated DNA and RNA sequences of the same tissues to construct a customized proteogenomic database that was appended with sequence variants from individual samples. Following that, the cellular immunogenicity of the detected epitopes is validated, and cancer vaccine and adoptive T-cell transfer personalized immunotherapies can be developed. Results obtained from this proteogenomic pipeline allowed her to identify actionable tumor-associated antigens and tumor-specific neoantigens, as well as to enhance the performance of *in silico* HLA prediction algorithm (24, 25). Recently, her lab also expanded the proteogenomic workflow to incorporate noncanonical sequences, originally considered as nonprotein-coding, for example, noncoding RNAs, the 3′ and 5′ untranslated regions and transposon elements. This new pipeline employed exome sequencing, RNA-seq, and RIBO-seq; and the resulting personalized database contains sequences from three-frame translation of RNAs (26). To deal with the inflated database size and thus FDR, her lab developed a new search engine called NewAnce that combined MaxQuant and COMET. Besides, RIBO-seq data was also used to detect the actual translatome so that a smaller and more precise customized database can be built to reduce FDR (Fig. 4).

Intense discussion followed this talk. Prof. Yasushi Ishihama was especially curious about the selectivity of the method for purifying HLA peptides, while another participant was extremely interested if the inclusion of noncanonical peptides and noncoding peptides improved antigen target identification. Another question pertains to the most important parameters in the AI algorithm used for antigen discovery. Dr Bassani-Sternberg was very impressed with the specificity (up to 95%) of the purification method that relies on pan-HLA antibodies. As of now, noncanonical peptide sequences have not been included for clinical studies and Dr Bassani-Sternberg also found little immunogenicity among them; and she added that the three most important pieces of information to consider in developing the AI algorithm are the detection of mutation and expression levels with RNA-seq and the binding affinities of HLA peptides.

## Future Perspective

The second AOHUPO OES was very well-received especially among the early career researchers. During the postmortem of the event, several proposals have been put forward, including: (i) to encourage AO young scientists to organize the events themselves, (ii) to split the event into separate theoretical and lab demo sessions, as well as (iii) to rotate the organizers among the AO regions. The AOHUPO decided that this event should be continued and should remain online even though the threat from COVID-19 had subsided, since it is an excellent platform that brings AO researchers together for knowledge sharing. Meanwhile, the AOHUPO also organizes a biennial on-site event called the AOHUPO Congress. In 2023, the Joint 11th AOHUPO and seventh AOAPO (Asia Oceania Agricultural Proteomics Organization) will be co-organized by the Singapore Society of Mass Spectrometry (SSMS) and the Malaysian Proteomics Society (MAPS). The Congress will take place in Singapore on 8th-10th May 2023 (https://www.aohupo-aoapo-2023.org/). We are looking forward to the first successful face-to-face AOHUPO Congress since the COVID-19 pandemic.

## Conflict of interest

The authors declare that they have no conflict of interest with the contents of this article.
